# Crystal structure and Hirshfeld surface analysis of 8-benzyl-1-[(4-methyl­phen­yl)sulfon­yl]-2,7,8,9-tetra­hydro-1*H*-3,6:10,13-diep­oxy-1,8-benzodi­aza­cyclo­penta­decine ethanol hemisolvate

**DOI:** 10.1107/S2056989024002275

**Published:** 2024-03-26

**Authors:** Gleb M. Burkin, Elizaveta A. Kvyatkovskaya, Victor N. Khrustalev, Khudayar I. Hasanov, Nurlana D. Sadikhova, Mehmet Akkurt, Ajaya Bhattarai

**Affiliations:** a RUDN University, 6 Miklukho-Maklaya St., Moscow 117198, Russian Federation; b Zelinsky Institute of Organic Chemistry of RAS, 4, 7 Leninsky Prospect, 119991 Moscow, Russian Federation; c Western Caspian University, Istiqlaliyyat Street 31, AZ1001, Baku, Azerbaijan; d Azerbaijan Medical University, Scientific Research Centre (SRC), A. Kasumzade St. 14. Baku, AZ 1022, Azerbaijan; e Department of Chemistry, Baku State University, Z. Xalilov Str. 23, Az 1148 Baku, Azerbaijan; f Department of Physics, Faculty of Sciences, Erciyes University, 38039 Kayseri, Türkiye; g Department of Chemistry, M.M.A.M.C (Tribhuvan University), Biratnagar, Nepal; Katholieke Universiteit Leuven, Belgium

**Keywords:** crystal structure, furan, sulfonamide, macrocycles, hydrogen bonds, C—H⋯π inter­actions, Hirshfeld surface analysis

## Abstract

The asymmetric unit of the title compound, 2C_31_H_28_N_2_O_4_S·C_2_H_6_O, contains a parent mol­ecule and a half mol­ecule of ethanol solvent. The main compound stabilizes its mol­ecular conformation by forming a ring with an *R*_1_^2^(7) motif with the ethanol solvent mol­ecule. In the crystal, mol­ecules are connected by C—H⋯O and O—H⋯O hydrogen bonds, forming a three-dimensional network. In addition, C—H⋯π inter­actions also strengthen the mol­ecular packing.

## Abstract 2

In the crystal, mol­ecules are connected by C—H⋯O and O—H⋯O hydrogen bonds, forming a three-dimensional network. In addition, C—H⋯π inter­actions also strengthen the mol­ecular packing.

## 1. Chemical context

**Figure 1:**
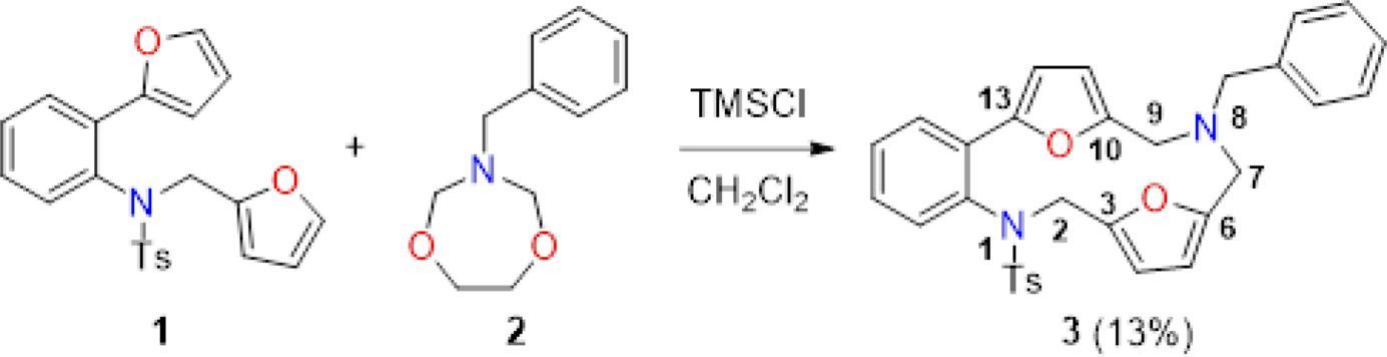
Synthesis of 8-benzyl-1-[(4-methyl­phen­yl)sulfon­yl]-2,7,8,9-tetra­hydro-1*H*-3,6:10,13-diep­oxy-1,8-benzodi­aza­cyclo­penta­decine.

Inter­molecular weak inter­actions play critical roles in maintaining supra­molecular networks with diverse structures and functions, wherein multiple weak bonds can cooperate to promote both the formation and stabilization of the assemblies (Aliyeva *et al.*, 2024 ▸). *N*-Ligands bearing amino and imino moieties provide a rich coordination chemistry (Kopylovich *et al.*, 2011*a* ▸,*b* ▸,*c* ▸; Mahmudov *et al.*, 2013 ▸, 2021 ▸). A number of metal complexes with *N-*ligands have been reported and characterized (Mahmoudi *et al.*, 2017*a* ▸,*b* ▸); some of them possess inter­esting spectroscopic, supra­molecular and catalytic properties (Akbari Afkhami *et al.*, 2017 ▸; Gurbanov *et al.*, 2018 ▸, 2020 ▸). Similarly to the design of *N*-heterocycles (Abdelhamid *et al.*, 2011 ▸; Khalilov *et al.*, 2021 ▸; Safavora *et al.*, 2019 ▸), particular attention has also been paid to the decoration of the secondary coordination sphere of metal complexes (Gurbanov *et al.*, 2022*a* ▸,*b* ▸; Mahmoudi *et al.*, 2019 ▸, 2021 ▸). Depending on the attached functional groups, the chemical properties of *N*-heterocyclic ligands and their metal complexes can be improved (Aliyeva *et al.*, 2024 ▸). On the other hand, macrocyclic structures containing furan fragments have been described in the literature: furan-containing crown ethers and porphyrinoids (Märkl *et al.*, 1997 ▸), furan-containing porphyrins (Srinivasan *et al.*, 1997 ▸), cyclic oligomers of furane-containing amino acids (Chakraborty *et al.*, 2007 ▸) and anti­aromatic macrocycles in which furan blocks are inter­connected through diene elements (Märkl *et al.*, 1996 ▸). Materials based on macrocycles have applications in drug discovery, can be used for the separation of isomers and metals, purification of organic solvents, chemical detection systems *etc.* Continuing our research into the chemistry of furyl-substituted sulfonamides (Guliyeva *et al.*, 2024 ▸; Mammadova *et al.*, 2023 **a* ▸,b* ▸; Borisova *et al.*, 2018 **a* ▸,b* ▸), a new approach toward the synthesis of difuryl-substituted arenesulfonamide macrocycles has been developed. The synthetic procedure is the Mannich reaction between a difuryl-substituted toluene­sulfonamide **1** and 3-benzyl-1,5,3-dioxazepane **2** under Lewis acid catalysis (Fig. 1 ▸). Tri­methyl­silyl chloride is the most efficient catalyst and has exhibited satisfactory results. 
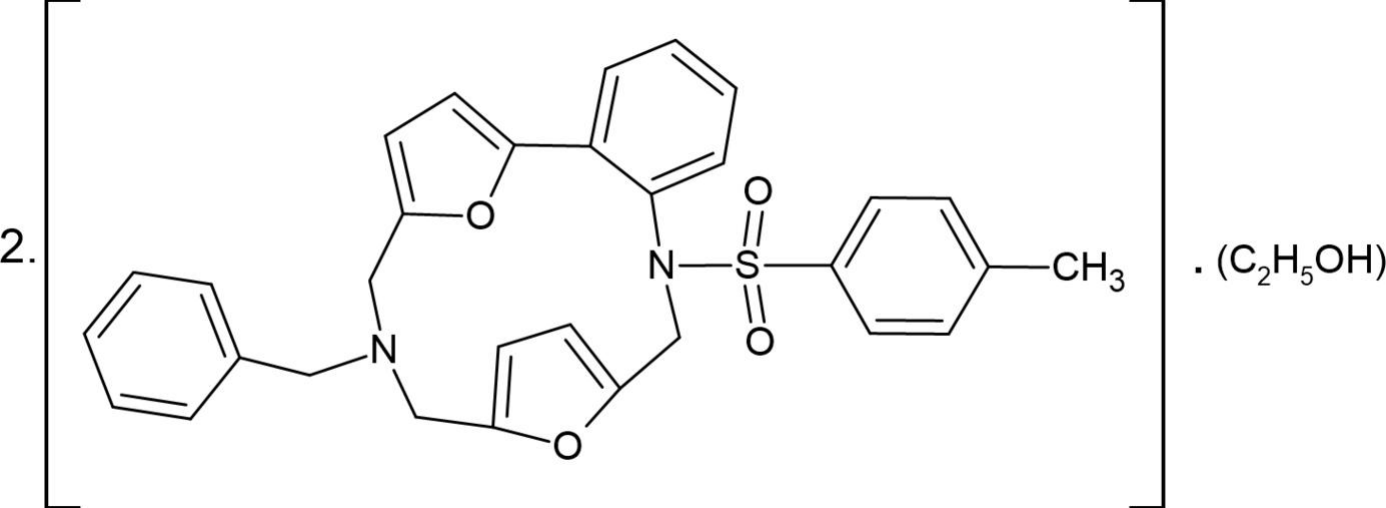


## 2. Structural commentary

**Figure 2:**
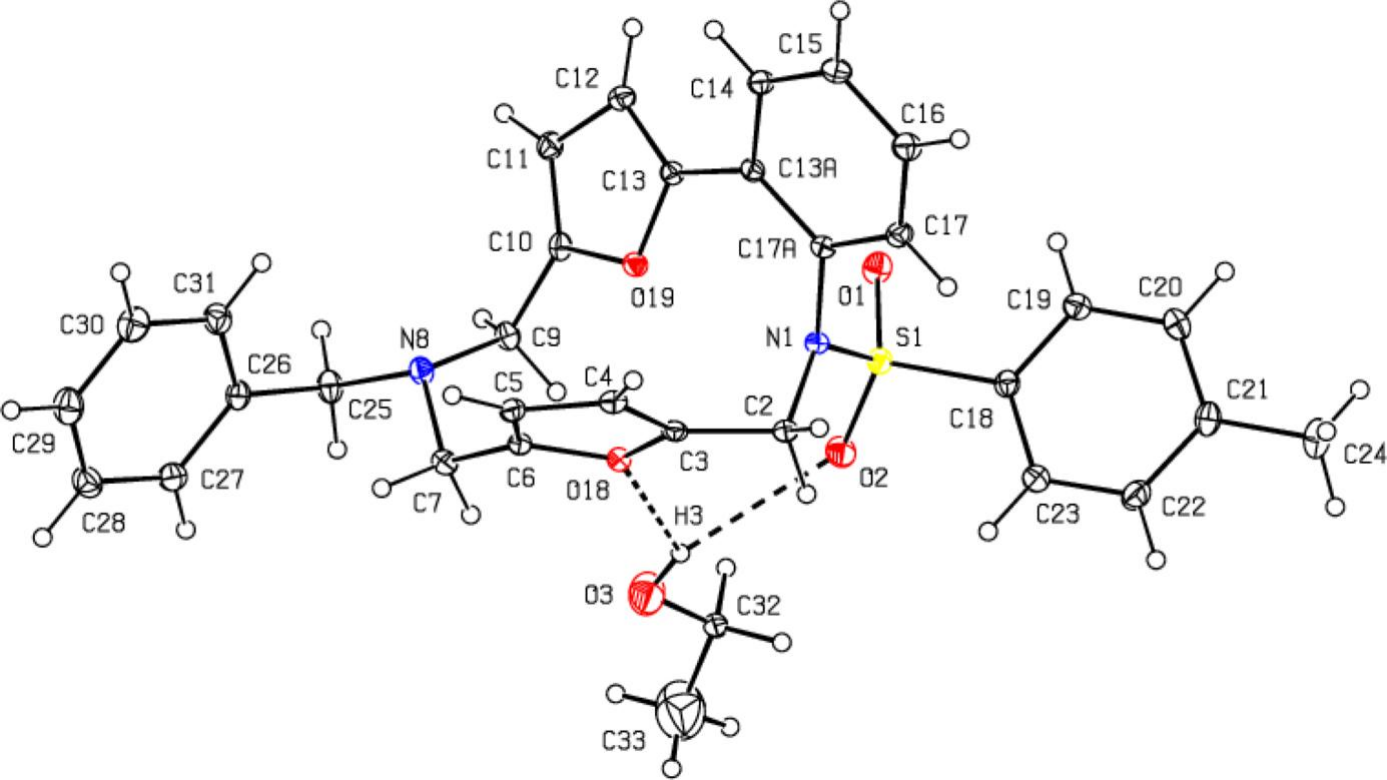
Mol­ecular structure of the title compound showing the atom labelling and ellipsoids at the 30% probability level with hydrogen bonds indicated by dashed lines.

The asymmetric unit of the title compound contains a parent mol­ecule and a half mol­ecule of the solvent ethanol. The main compound stabilizes its mol­ecular conformation by forming a ring with an 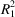 (7) motif with the ethanol solvent mol­ecule (Fig. 2 ▸; Bernstein *et al.*, 1995 ▸). While the two furan rings (O18/C3–C6 and O19/C10–C13) in the central ring system subtend an angle of 75.50 (7)° with each other, they make dihedral angles of 50.15 (7) and 25.58 (7)°, respectively, with the benzene ring (C13*A*/C14–C17/C17*A*) in the same central ring. The phenyl (C26–C31) and benzene (C18–C23) rings outside the central ring make an angle of 65.91 (8)° with each other, and subtend dihedral angles of 68.95 (7) and 48.71 (7)°, respectively, with the benzene ring (C13*A*/C14–C17/C17*A*) in the central ring. The r.m.s. deviations of the planes fitted through the atoms attached to N1 and N8 are 0.0744 and 0.1889 Å, respectively, with the distances of N1 and N8 to these planes being 0.1288 (8) and 0.3271 (10) Å, respectively. The sums of the angles around the central atoms N1 and N8 are 356.20 and 334.96°, respectively. As can be seen, N1 is closer to the plane of neighboring atoms than N8, and the sum of the angles around it is closer to 360°. The S1 atom bonded to the N1 atom causes it to have a more planar environment. In the title compound, the N atoms are located on opposite sides of the mean plane through the thirteen-membered difuryl-containing ring. Bond length and angle values in the title compound are comparable to those in the related compounds discussed in the *Database survey* (section 4).

## 3. Supra­molecular features and Hirshfeld surface analysis

**Figure 3:**
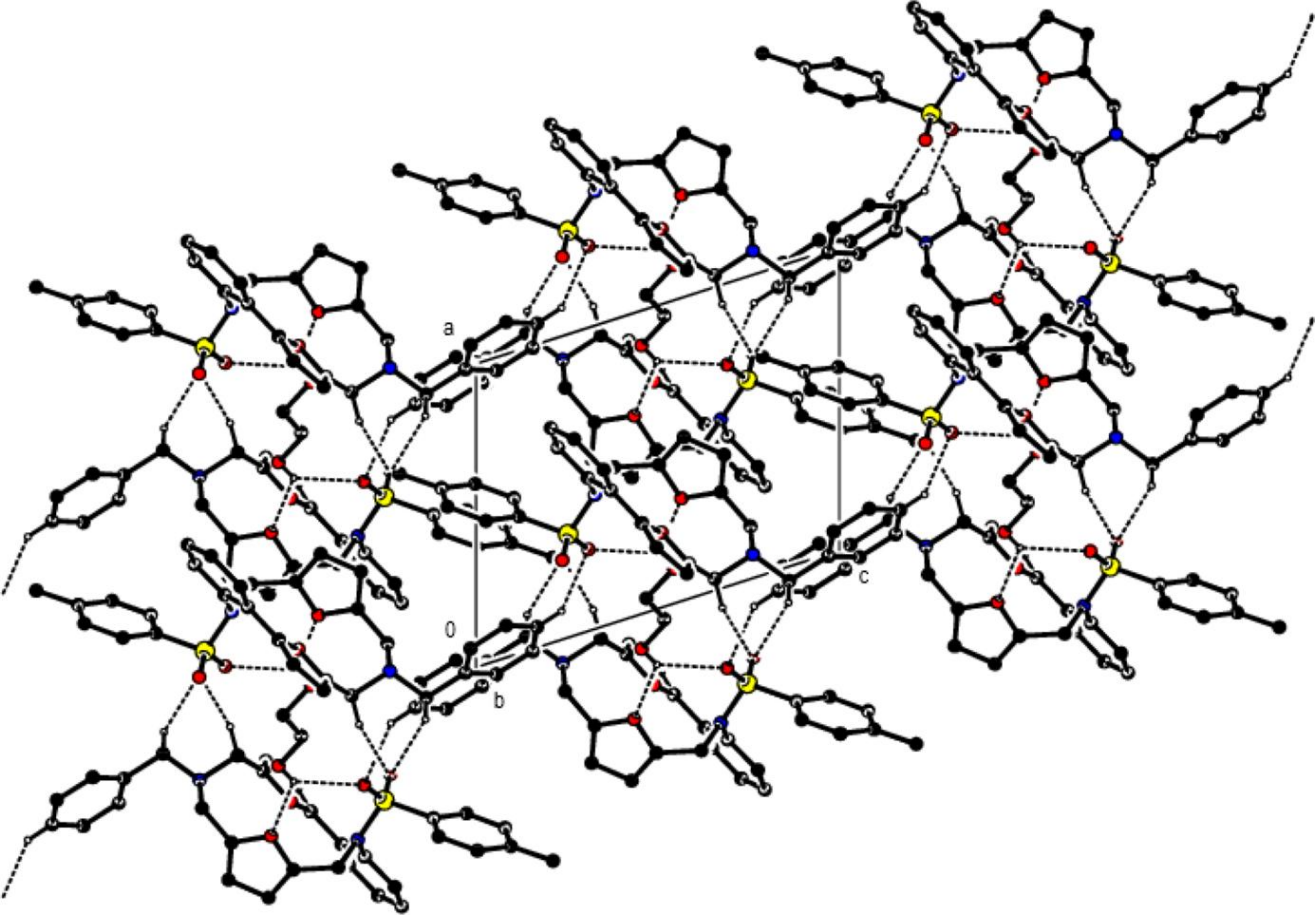
Crystal packing viewed along the *b*-axis showing C—H⋯O and O—H⋯O hydrogen bonds shown as dashed lines.

**Figure 4:**
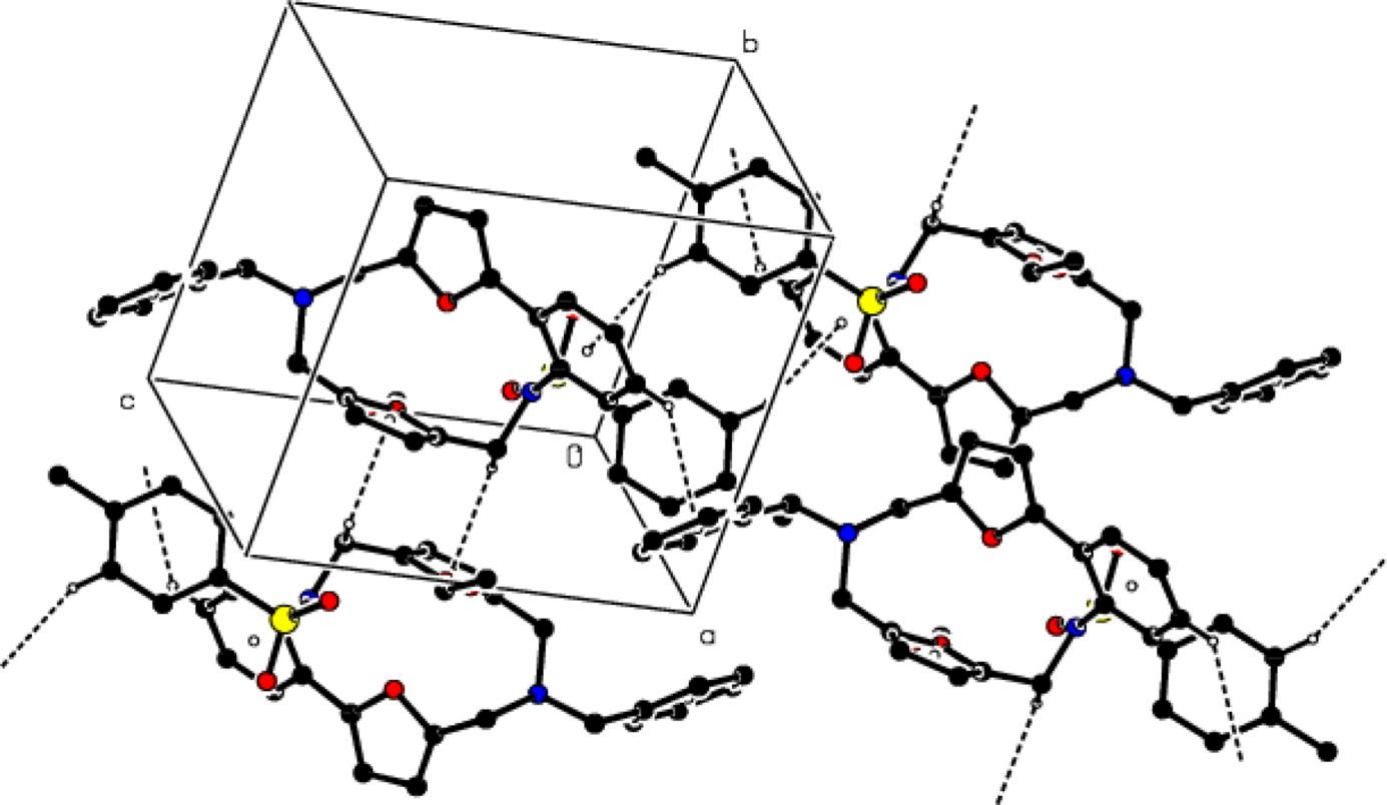
View of the C—H⋯π inter­actions in the crystal packing, shown as dashed lines.

In the crystal, mol­ecules are connected by C—H⋯O and O—H⋯O hydrogen bonds, forming a three-dimensional network (Table 1 ▸; Fig. 3 ▸). In addition, C—H⋯π inter­actions (Table 1 ▸) also strengthen the mol­ecular packing (Fig. 4 ▸).

**Figure 5:**
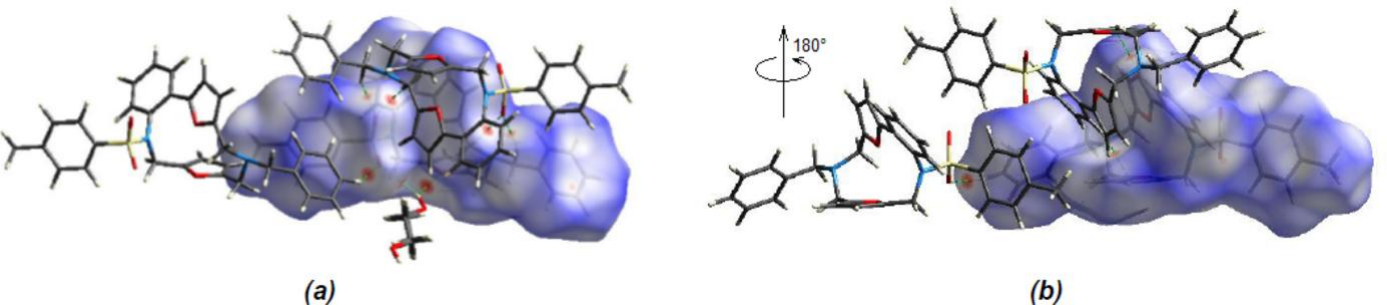
Front (*a*) and back (*b*) views of the three-dimensional Hirshfeld surface, showing some C—H⋯O and O—H⋯O hydrogen bonds.

**Figure 6:**
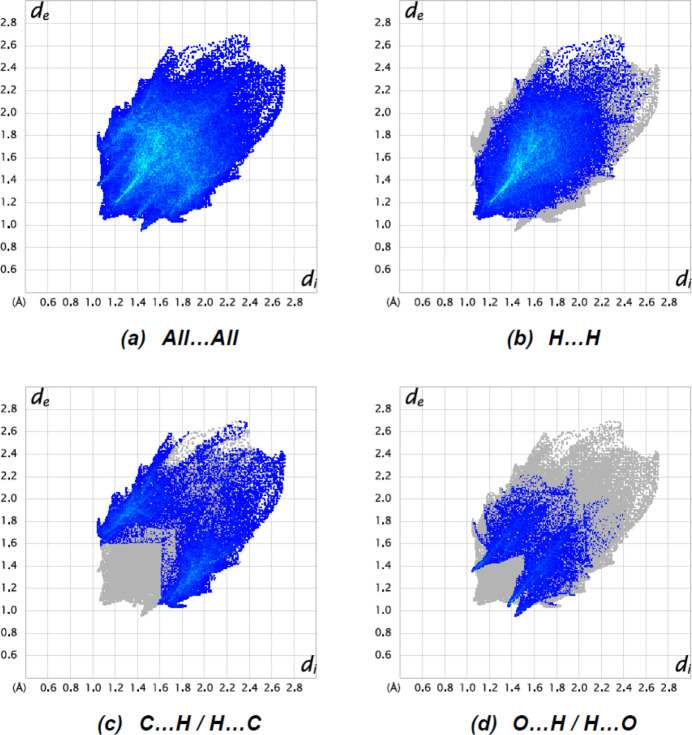
The two-dimensional fingerprint plots for the title mol­ecule showing (*a*) all inter­actions, and delineated into (*b*) H⋯H, (*c*) C⋯H/H⋯C and (*d*) O⋯H/H⋯O inter­actions. The *d*_ĩ_ and *d*_e_ values are the closest inter­nal and external distances (in Å) from given points on the Hirshfeld surface.

Two-dimensional fingerprints and the Hirshfeld surface of the title mol­ecule were computed using *CrystalExplorer17.5* (Spackman *et al.*, 2021 ▸). The Hirshfeld surface was mapped over *d*_norm_ in the range −0.1635 (red) to +1.5099 (blue) a.u. (Fig. 5 ▸). The overall two-dimensional fingerprint plot and those delineated into H⋯H, C⋯H/H⋯C and O⋯H/H⋯O contacts are illustrated in Fig. 5 ▸ *a*–*d*, respectively. The pairs of spikes with tips at *d*_e_ + *d*_i_ = 2.62 Å in Fig. 6 ▸ *c* and at *d*_e_ + *d*_i_ = 2.40 Å in Fig. 6 ▸ *d* indicate weak hydrogen-bonding inter­actions. The most significant contributions to the Hirshfeld surface are from H⋯H (56.6%, Fig. 6 ▸ *b*), C⋯H/H⋯C (26.6%, Fig. 6 ▸ *c*) and O⋯H/H⋯O (13.9%, Fig. 6 ▸ *d*) inter­actions, indicating that the highest contributions arise from contacts in which H atoms are involved. Except for C⋯C inter­actions (2.1%), the other contributions are less than 1.5%.

## 4. Database survey

A search of the Cambridge Structural Database (CSD, Version 5.42, update of September 2021; Groom *et al.*, 2016 ▸) found the compounds most similar to the title compound to be 7,14,16-trimethyl-17-(tri­fluoro­acet­yl)-18,19-dioxa-7,17-di­aza­tetra­cyclo­[11.3.1.1^2,5^.1^9,12^]nona­deca-2,4,9,11-tetraen-15-one (CSD refcode YEYXAF; Yıldırım *et al.*, 2023 ▸) and 1,8,12,19,24,26-hexa­aza­penta­cyclo­[17.3.1.1^3,6^.1^8, 12^.1^14,17^]hexa­cosa-3,5,14,16-tetra­ene ethyl acetate solvate dihydrate (NOYCOW; Jana *et al.*, 2019 ▸).

NOYCOW crystallizes in the monoclinic space group *I*2/*a* with *Z* = 8 while YEYXAF crystallizes in the ortho­rhom­bic space group *Pbca* with *Z* = 8. The furan rings in YEYXAF are nearly perpendicular to the mean plane through the main twelve-membered difuryl-containing ring and their oxygen atoms are oriented towards opposite sides. In NOYCOW, the pyrrole rings are also almost perpendicular to the sixteen-membered ring, but the two pyrrolic NH atoms are oriented in the same direction.

In the title compound, the N atoms are located on either side of the mean plane through the thirteen-membered difuryl-containing ring. The phenyl group of the title mol­ecule is approximately parallel to this thirteen-membered ring, and the benzene ring attached to the S atom is also approximately parallel.

## 5. Synthesis and crystallization

The starting materials *N*-(2-(furan-2-yl)phen­yl)-*N*-(furan-2-yl­meth­yl)-4-methyl­benzene­sulfonamide **1** (100 mg, 0.25 mmol) and 3-benzyl-1,5,3-dioxazepane **2** (52 mg, 0.27 mmol) in 5 mL of DCM were placed into a two-neck flask. The reaction mixture was purged with argon for 10 min under stirring and cooling in an ice–water bath. Chloro­tri­methyl­silane (TMSCl, 0.11 mL, 0.84 mmol) was added to the reaction with stirring at 273 K. After the addition, the reaction mixture was stirred for 24 h under argon. Then a saturated Na_2_CO_3_ solution was added to the reaction mixture to adjust the pH to ∼7. Then it was poured into water (20 mL) and extracted with DCM (3 × 10 mL). The reaction product was purified by column chromatography (SiO_2_, 20 × 1.1 cm, eluent: hepta­ne/ethyl acetate 10:1, TLC: heptane/ethyl acetate 4:1). The title compound was obtained as a colorless powder, yield 13%, 17 mg (0.032 mmol); m.p. > 523 K (with decomp.). Single crystals of the title compound were grown from EtOH. IR (KBr), *ν* (cm^−1^): 1348 (ν_as_ SO_2_), 1162 (ν_s_ SO_2_). ^1^H NMR (700.2 MHz, CDCl_3_) (*J*, Hz): *δ* 7.47 (*d*, *J* = 8.1 Hz, 2H), 7.33 (*d*, *J* = 8.1 Hz, 1H), 7.27-7.20 (*m*, 7H), 7.13 (*d*, *J* = 8.1 Hz, 2H), 6.98 (*d*, *J* = 7.9 Hz, 1H), 6.30 (*d*, *J* = 2.9 Hz, 1H), 5.89 (*d*, *J* = 2.9 Hz, 1H), 5.74 (*d*, *J* = 2.9 Hz, 1H), 5.62 (*d*, *J* = 2.9 Hz, 1H), 5.15 (*d*, *J* = 14.8 Hz, 1H), 4.10 (*d*, *J* = 14.8 Hz, 1H), 3.86 (*s*, 2H), 3.79 (*d*, *J* = 15.0 Hz, 1H), 3.65 (*d*, *J* = 15.0 Hz, 1H), 3.57 (*d*, *J* = 13.8 Hz, 1H), 3.47 (*d*, *J* = 13.8 Hz, 1H), 2.33 (*s*, 3H). ^13^C{^1^H} NMR (176.1 MHz, CDCl_3_): *δ* 152.3, 152.1, 150.8, 146.4, 142.1, 138.2, 135.9, 134.9, 132.8, 128.9, 128.8, 128.3 (2C), 127.9 (2C), 127.7, 127.3 (2C), 126.7 (2C), 126.0, 114.1, 110.5, 109.6, 108.1, 107.0, 54.8, 49.7, 48.9, 48.4, 20.5. MS (ESI) *m*/*z*: [*M* + H]^+^ 525. Elemental analysis calculated (%) for C_31_H_28_N_2_O_4_S: C 70.97, H 5.38, N 5.34, S 6.11; found: C 71.11, H 5.49, N 5.59, S 5.87.

## 6. Refinement

Crystal data, data collection and structure refinement details are summarized in Table 2 ▸. All C-bound H atoms were positioned geometrically (C—H = 0.95 and 0.99 Å) and refined using a riding model with *U*_iso_(H) = 1.2 or 1.5*U*_eq_(C). The O-bound H atom of the ethanol solvent was located in difference-Fourier maps [O3—H3 = 0.88 (6) Å] and refined freely with *U*_iso_(H) = 1.5*U*_eq_(O). The site occupation factors of the solvent atoms were fixed at 0.5.

## Supplementary Material

Crystal structure: contains datablock(s) I. DOI: 10.1107/S2056989024002275/vm2297sup1.cif

Structure factors: contains datablock(s) I. DOI: 10.1107/S2056989024002275/vm2297Isup2.hkl

CCDC reference: 2338754

Additional supporting information:  crystallographic information; 3D view; checkCIF report

## Figures and Tables

**Table 1:** Hydrogen-bond geometry (Å, °) *Cg*1, *Cg*3 and *Cg*5 are the centroids of the O18/C3–C6 furan, C13*A*/C14–C17/C17*A* benzene and C26–C31 phenyl rings, respectively.

*D*—H⋯*A*	*D*—H	H⋯*A*	*D*⋯*A*	*D*—H⋯*A*
C9—H9*A*⋯O1^i^	0.99	2.53	3.4057 (17)	148
C25—H25*B*⋯O1^i^	0.99	2.59	3.5125 (18)	155
O3—H3⋯O2	0.88 (7)	2.52 (6)	3.251 (3)	141 (6)
O3—H3⋯O18	0.88 (7)	2.47 (7)	3.097 (4)	129 (5)
C2—H2*A*⋯*Cg*1^ii^	0.99	2.56	3.2916 (12)	131
C16—H16⋯*Cg*5^iii^	0.95	2.80	3.5220 (15)	133
C20—H20⋯*Cg*3^iv^	0.95	2.83	3.5707 (15)	135

Symmetry codes: (i) 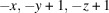 ; (ii) 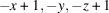 ; (iii) 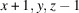 ; (iv) 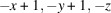 .

**Table 2:** Experimental details

Crystal data	
Chemical formula	2C_31_H_28_N_2_O_4_S·C_2_H_6_O
*M*_r_	1095.29
Crystal system, space group	Triclinic, *P* 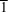
Temperature (K)	100
*a*, *b*, *c* (Å)	10.7108 (2), 11.7817 (2), 12.9233 (3)
α, β, γ (°)	73.831 (2), 67.667 (2), 67.380 (2)
*V* (Å^3^)	1375.62 (6)
*Z*	1
Radiation type	Cu *K*α
μ (mm^−1^)	1.39
Crystal size (mm)	0.28 × 0.25 × 0.21

Data collection	
Diffractometer	XtaLAB Synergy, Dualflex, HyPix
Absorption correction	Multi-scan (*CrysAlis PRO*; Rigaku OD, 2021 ▸)
*T*_min_, *T*_max_	0.696, 0.759
No. of measured, independent and observed [*I* > 2σ(*I*)] reflections	41981, 5824, 5577
*R*_int_	0.035
(sin θ/λ)_max_ (Å^−1^)	0.634

Refinement	
*R*[*F* ^2^ > 2σ(*F* ^2^)], *wR*(*F* ^2^), *S*	0.034, 0.088, 1.06
No. of reflections	5824
No. of parameters	375
H-atom treatment	H atoms treated by a mixture of independent and constrained refinement
Δρ_max_, Δρ_min_ (e Å^−3^)	0.36, −0.38

Computer programs: *CrysAlis PRO* (Rigaku OD, 2021 ▸), *SHELXT2016/6* (Sheldrick, 2015*a* ▸), *SHELXL2016/6* (Sheldrick, 2015*b* ▸, *ORTEP-3 for Windows* (Farrugia, 2012 ▸) and *PLATON* (Spek, 2020 ▸).

## supplementary crystallographic information

### Crystal data

**Table:**

2C_31_H_28_N_2_O_4_S·C_2_H_6_O	*Z* = 1
*M**_r_* = 1095.29	*F*(000) = 578
Triclinic, *P*1	*D*_x_ = 1.322 Mg m^−^^3^
*a* = 10.7108 (2) Å	Cu *K*α radiation, λ = 1.54184 Å
*b* = 11.7817 (2) Å	Cell parameters from 29573 reflections
*c* = 12.9233 (3) Å	θ = 3.7–77.3°
α = 73.831 (2)°	µ = 1.39 mm^−^^1^
β = 67.667 (2)°	*T* = 100 K
γ = 67.380 (2)°	Prism, colourless
*V* = 1375.62 (6) Å^3^	0.28 × 0.25 × 0.21 mm

### Data collection

**Table:**

XtaLAB Synergy, Dualflex, HyPix diffractometer	5577 reflections with *I* > 2σ(*I*)
Radiation source: micro-focus sealed X-ray tube	*R*_int_ = 0.035
φ and ω scans	θ_max_ = 77.9°, θ_min_ = 3.7°
Absorption correction: multi-scan (CrysAlisPro; Rigaku OD, 2021)	*h* = −13→13
*T*_min_ = 0.696, *T*_max_ = 0.759	*k* = −14→13
41981 measured reflections	*l* = −16→15
5824 independent reflections	

### Refinement

**Table:**

Refinement on *F*^2^	Hydrogen site location: mixed
Least-squares matrix: full	H atoms treated by a mixture of independent and constrained refinement
*R*[*F*^2^ > 2σ(*F*^2^)] = 0.034	*w* = 1/[σ^2^(*F*_o_^2^) + (0.0392*P*)^2^ + 0.6403*P*] where *P* = (*F*_o_^2^ + 2*F*_c_^2^)/3
*wR*(*F*^2^) = 0.088	(Δ/σ)_max_ < 0.001
*S* = 1.06	Δρ_max_ = 0.36 e Å^−^^3^
5824 reflections	Δρ_min_ = −0.38 e Å^−^^3^
375 parameters	Extinction correction: SHELXL-2019/3 (Sheldrick, 2015b), Fc^*^=kFc[1+0.001xFc^2^λ^3^/sin(2θ)]^-1/4^
0 restraints	Extinction coefficient: 0.0016 (2)

### Special details

**Table:**

Geometry. All esds (except the esd in the dihedral angle between two l.s. planes) are estimated using the full covariance matrix. The cell esds are taken into account individually in the estimation of esds in distances, angles and torsion angles; correlations between esds in cell parameters are only used when they are defined by crystal symmetry. An approximate (isotropic) treatment of cell esds is used for estimating esds involving l.s. planes.

### Fractional atomic coordinates and isotropic or equivalent isotropic displacement parameters (Å^2^)

**Table:**

	*x*	*y*	*z*	*U*_iso_*/*U*_eq_	Occ. (<1)
S1	0.34319 (3)	0.29237 (3)	0.25323 (2)	0.01869 (9)	
O1	0.26473 (9)	0.42189 (9)	0.23711 (8)	0.0254 (2)	
O2	0.27094 (10)	0.20120 (9)	0.30881 (8)	0.0275 (2)	
N1	0.44474 (10)	0.27652 (9)	0.32641 (8)	0.0172 (2)	
C2	0.51453 (13)	0.15058 (10)	0.37966 (10)	0.0191 (2)	
H2A	0.485159	0.088176	0.364704	0.023*	
H2B	0.619013	0.129923	0.346117	0.023*	
C3	0.47475 (12)	0.14584 (10)	0.50358 (10)	0.0175 (2)	
C4	0.54024 (13)	0.15296 (11)	0.57161 (10)	0.0198 (2)	
H4	0.634721	0.154918	0.550100	0.024*	
C5	0.43909 (13)	0.15696 (11)	0.68239 (10)	0.0199 (2)	
H5	0.453219	0.162182	0.748959	0.024*	
C6	0.31931 (13)	0.15184 (10)	0.67389 (10)	0.0185 (2)	
C7	0.17501 (13)	0.15988 (11)	0.75523 (10)	0.0220 (2)	
H7A	0.131541	0.114502	0.731987	0.026*	
H7B	0.183651	0.118517	0.831302	0.026*	
N8	0.08088 (11)	0.28896 (10)	0.76194 (8)	0.0200 (2)	
C9	0.04134 (13)	0.35259 (11)	0.65683 (10)	0.0204 (2)	
H9A	−0.061979	0.397627	0.677668	0.024*	
H9B	0.060752	0.289099	0.611373	0.024*	
C10	0.11988 (12)	0.44237 (11)	0.58612 (10)	0.0189 (2)	
C11	0.09010 (13)	0.56626 (12)	0.57983 (10)	0.0223 (3)	
H11	0.006365	0.620768	0.622820	0.027*	
C12	0.20853 (13)	0.59902 (11)	0.49635 (11)	0.0221 (2)	
H12	0.219159	0.679579	0.473175	0.027*	
C13	0.30360 (12)	0.49285 (11)	0.45610 (10)	0.0173 (2)	
C13A	0.44410 (12)	0.46902 (10)	0.37101 (10)	0.0170 (2)	
C14	0.52046 (13)	0.55094 (11)	0.35073 (10)	0.0197 (2)	
H14	0.480254	0.616767	0.394083	0.024*	
C15	0.65231 (13)	0.53900 (11)	0.26973 (11)	0.0223 (2)	
H15	0.701290	0.595894	0.258244	0.027*	
C16	0.71286 (13)	0.44340 (12)	0.20519 (11)	0.0238 (3)	
H16	0.802673	0.435140	0.148619	0.029*	
C17	0.64055 (13)	0.36040 (11)	0.22444 (10)	0.0210 (2)	
H17	0.681655	0.294797	0.180736	0.025*	
C17A	0.50862 (12)	0.37150 (10)	0.30676 (10)	0.0169 (2)	
O18	0.33994 (9)	0.14221 (7)	0.56487 (7)	0.01775 (17)	
O19	0.25043 (9)	0.39580 (7)	0.51033 (7)	0.01831 (18)	
C18	0.45360 (13)	0.25328 (11)	0.11787 (10)	0.0194 (2)	
C19	0.47466 (14)	0.34721 (12)	0.02676 (11)	0.0231 (3)	
H19	0.429460	0.432036	0.036840	0.028*	
C20	0.56225 (14)	0.31628 (13)	−0.07921 (11)	0.0256 (3)	
H20	0.577191	0.380592	−0.141437	0.031*	
C21	0.62851 (13)	0.19256 (13)	−0.09572 (11)	0.0248 (3)	
C22	0.60615 (16)	0.09972 (13)	−0.00297 (12)	0.0298 (3)	
H22	0.651317	0.014844	−0.012877	0.036*	
C23	0.51916 (15)	0.12903 (12)	0.10348 (11)	0.0273 (3)	
H23	0.504526	0.064868	0.165925	0.033*	
C24	0.72086 (16)	0.15961 (15)	−0.21168 (12)	0.0332 (3)	
H24A	0.694267	0.229732	−0.269594	0.050*	
H24B	0.707489	0.086191	−0.221990	0.050*	
H24C	0.820799	0.141504	−0.218776	0.050*	
C25	−0.04484 (13)	0.29217 (13)	0.86246 (11)	0.0241 (3)	
H25A	−0.089738	0.232837	0.862308	0.029*	
H25B	−0.114917	0.376593	0.859809	0.029*	
C26	−0.00487 (12)	0.25859 (12)	0.96982 (10)	0.0207 (2)	
C27	−0.04248 (13)	0.16461 (12)	1.05579 (11)	0.0240 (3)	
H27	−0.093326	0.119180	1.046874	0.029*	
C28	−0.00605 (15)	0.13668 (13)	1.15487 (12)	0.0290 (3)	
H28	−0.033377	0.073309	1.213575	0.035*	
C29	0.06986 (15)	0.20120 (14)	1.16770 (12)	0.0318 (3)	
H29	0.095792	0.181438	1.234727	0.038*	
C30	0.10807 (15)	0.29490 (14)	1.08238 (12)	0.0306 (3)	
H30	0.159859	0.339513	1.091183	0.037*	
C31	0.07072 (14)	0.32339 (13)	0.98440 (11)	0.0258 (3)	
H31	0.096909	0.387811	0.926505	0.031*	
O3	0.1154 (3)	0.0518 (3)	0.5446 (2)	0.0509 (6)	0.5
H3	0.183 (6)	0.085 (5)	0.503 (5)	0.076*	0.5
C32	0.0470 (6)	0.0434 (4)	0.4684 (5)	0.0246 (10)	0.5
H32A	−0.011759	0.128074	0.444417	0.030*	0.5
H32B	0.121887	0.012197	0.399757	0.030*	0.5
C33	−0.0412 (13)	−0.0361 (10)	0.5180 (10)	0.093 (4)	0.5
H33A	−0.082799	−0.036983	0.462603	0.140*	0.5
H33B	0.016411	−0.120799	0.540200	0.140*	0.5
H33C	−0.117428	−0.004755	0.584925	0.140*	0.5

### Atomic displacement parameters (Å^2^)

**Table:**

	*U*^11^	*U*^22^	*U*^33^	*U*^12^	*U*^13^	*U*^23^
S1	0.01708 (14)	0.02084 (16)	0.01848 (15)	−0.00704 (11)	−0.00430 (11)	−0.00365 (11)
O1	0.0207 (4)	0.0262 (5)	0.0259 (5)	−0.0012 (4)	−0.0080 (4)	−0.0067 (4)
O2	0.0265 (5)	0.0350 (5)	0.0263 (5)	−0.0196 (4)	−0.0040 (4)	−0.0042 (4)
N1	0.0208 (5)	0.0145 (5)	0.0166 (5)	−0.0065 (4)	−0.0056 (4)	−0.0018 (4)
C2	0.0227 (6)	0.0136 (5)	0.0188 (6)	−0.0043 (4)	−0.0053 (5)	−0.0027 (4)
C3	0.0185 (5)	0.0114 (5)	0.0199 (6)	−0.0040 (4)	−0.0045 (4)	−0.0014 (4)
C4	0.0205 (6)	0.0153 (5)	0.0233 (6)	−0.0052 (4)	−0.0072 (5)	−0.0027 (4)
C5	0.0252 (6)	0.0155 (5)	0.0195 (6)	−0.0050 (4)	−0.0086 (5)	−0.0030 (4)
C6	0.0238 (6)	0.0131 (5)	0.0161 (5)	−0.0040 (4)	−0.0060 (4)	−0.0015 (4)
C7	0.0247 (6)	0.0193 (6)	0.0196 (6)	−0.0083 (5)	−0.0046 (5)	−0.0008 (4)
N8	0.0187 (5)	0.0216 (5)	0.0166 (5)	−0.0054 (4)	−0.0039 (4)	−0.0017 (4)
C9	0.0184 (5)	0.0234 (6)	0.0184 (6)	−0.0068 (5)	−0.0054 (4)	−0.0018 (5)
C10	0.0165 (5)	0.0226 (6)	0.0156 (5)	−0.0040 (4)	−0.0041 (4)	−0.0039 (4)
C11	0.0205 (6)	0.0212 (6)	0.0208 (6)	−0.0033 (5)	−0.0032 (5)	−0.0056 (5)
C12	0.0237 (6)	0.0173 (6)	0.0228 (6)	−0.0053 (5)	−0.0046 (5)	−0.0044 (5)
C13	0.0201 (6)	0.0163 (5)	0.0161 (5)	−0.0062 (4)	−0.0066 (4)	−0.0013 (4)
C13A	0.0186 (5)	0.0148 (5)	0.0160 (5)	−0.0039 (4)	−0.0067 (4)	−0.0002 (4)
C14	0.0227 (6)	0.0165 (5)	0.0205 (6)	−0.0059 (4)	−0.0077 (5)	−0.0027 (4)
C15	0.0225 (6)	0.0201 (6)	0.0262 (6)	−0.0100 (5)	−0.0078 (5)	−0.0013 (5)
C16	0.0185 (6)	0.0231 (6)	0.0264 (6)	−0.0074 (5)	−0.0021 (5)	−0.0045 (5)
C17	0.0203 (6)	0.0181 (6)	0.0222 (6)	−0.0048 (4)	−0.0039 (5)	−0.0051 (4)
C17A	0.0182 (5)	0.0147 (5)	0.0177 (5)	−0.0057 (4)	−0.0062 (4)	−0.0004 (4)
O18	0.0201 (4)	0.0168 (4)	0.0162 (4)	−0.0066 (3)	−0.0051 (3)	−0.0019 (3)
O19	0.0178 (4)	0.0160 (4)	0.0179 (4)	−0.0048 (3)	−0.0029 (3)	−0.0022 (3)
C18	0.0199 (5)	0.0222 (6)	0.0184 (6)	−0.0071 (5)	−0.0074 (4)	−0.0034 (4)
C19	0.0257 (6)	0.0201 (6)	0.0230 (6)	−0.0070 (5)	−0.0084 (5)	−0.0018 (5)
C20	0.0266 (6)	0.0291 (7)	0.0199 (6)	−0.0104 (5)	−0.0079 (5)	0.0012 (5)
C21	0.0211 (6)	0.0337 (7)	0.0203 (6)	−0.0057 (5)	−0.0086 (5)	−0.0064 (5)
C22	0.0380 (8)	0.0227 (6)	0.0261 (7)	−0.0025 (5)	−0.0115 (6)	−0.0076 (5)
C23	0.0377 (7)	0.0210 (6)	0.0216 (6)	−0.0080 (5)	−0.0100 (5)	−0.0017 (5)
C24	0.0290 (7)	0.0445 (8)	0.0224 (7)	−0.0046 (6)	−0.0076 (5)	−0.0101 (6)
C25	0.0185 (6)	0.0334 (7)	0.0197 (6)	−0.0099 (5)	−0.0034 (5)	−0.0041 (5)
C26	0.0168 (5)	0.0245 (6)	0.0181 (6)	−0.0045 (5)	−0.0029 (4)	−0.0056 (5)
C27	0.0223 (6)	0.0236 (6)	0.0253 (6)	−0.0075 (5)	−0.0054 (5)	−0.0049 (5)
C28	0.0307 (7)	0.0263 (7)	0.0251 (7)	−0.0065 (5)	−0.0094 (5)	0.0008 (5)
C29	0.0312 (7)	0.0378 (8)	0.0264 (7)	−0.0062 (6)	−0.0143 (6)	−0.0036 (6)
C30	0.0260 (7)	0.0394 (8)	0.0318 (7)	−0.0122 (6)	−0.0100 (6)	−0.0096 (6)
C31	0.0233 (6)	0.0308 (7)	0.0234 (6)	−0.0119 (5)	−0.0035 (5)	−0.0044 (5)
O3	0.0515 (15)	0.0704 (18)	0.0412 (13)	−0.0381 (14)	−0.0009 (11)	−0.0158 (12)
C32	0.032 (2)	0.0147 (16)	0.0178 (17)	−0.0037 (14)	−0.0020 (15)	−0.0027 (13)
C33	0.103 (8)	0.122 (8)	0.074 (7)	−0.060 (6)	−0.024 (6)	−0.014 (5)

### Geometric parameters (Å, º)

**Table:**

S1—O1	1.4309 (9)	C16—H16	0.9500
S1—O2	1.4339 (9)	C17—C17A	1.3923 (16)
S1—N1	1.6246 (10)	C17—H17	0.9500
S1—C18	1.7688 (12)	C18—C19	1.3880 (17)
N1—C17A	1.4435 (14)	C18—C23	1.3900 (18)
N1—C2	1.4843 (14)	C19—C20	1.3881 (18)
C2—C3	1.4829 (16)	C19—H19	0.9500
C2—H2A	0.9900	C20—C21	1.3915 (19)
C2—H2B	0.9900	C20—H20	0.9500
C3—C4	1.3517 (17)	C21—C22	1.3942 (19)
C3—O18	1.3688 (14)	C21—C24	1.5090 (18)
C4—C5	1.4300 (17)	C22—C23	1.3867 (19)
C4—H4	0.9500	C22—H22	0.9500
C5—C6	1.3534 (17)	C23—H23	0.9500
C5—H5	0.9500	C24—H24A	0.9800
C6—O18	1.3711 (14)	C24—H24B	0.9800
C6—C7	1.4869 (17)	C24—H24C	0.9800
C7—N8	1.4712 (15)	C25—C26	1.5101 (17)
C7—H7A	0.9900	C25—H25A	0.9900
C7—H7B	0.9900	C25—H25B	0.9900
N8—C25	1.4699 (15)	C26—C27	1.3930 (18)
N8—C9	1.4875 (15)	C26—C31	1.3948 (18)
C9—C10	1.4912 (16)	C27—C28	1.3952 (19)
C9—H9A	0.9900	C27—H27	0.9500
C9—H9B	0.9900	C28—C29	1.384 (2)
C10—C11	1.3544 (18)	C28—H28	0.9500
C10—O19	1.3725 (14)	C29—C30	1.389 (2)
C11—C12	1.4235 (17)	C29—H29	0.9500
C11—H11	0.9500	C30—C31	1.3859 (19)
C12—C13	1.3609 (17)	C30—H30	0.9500
C12—H12	0.9500	C31—H31	0.9500
C13—O19	1.3690 (14)	O3—C32	1.474 (7)
C13—C13A	1.4635 (16)	O3—H3	0.88 (6)
C13A—C17A	1.4041 (16)	C32—C33	1.438 (7)
C13A—C14	1.4045 (16)	C32—H32A	0.9900
C14—C15	1.3841 (17)	C32—H32B	0.9900
C14—H14	0.9500	C33—H33A	0.9800
C15—C16	1.3911 (18)	C33—H33B	0.9800
C15—H15	0.9500	C33—H33C	0.9800
C16—C17	1.3852 (17)		
			
O1—S1—O2	120.65 (6)	C17A—C17—H17	119.4
O1—S1—N1	106.82 (5)	C17—C17A—C13A	120.65 (11)
O2—S1—N1	106.39 (5)	C17—C17A—N1	117.53 (10)
O1—S1—C18	107.02 (6)	C13A—C17A—N1	121.77 (10)
O2—S1—C18	107.44 (6)	C3—O18—C6	106.71 (9)
N1—S1—C18	108.00 (5)	C13—O19—C10	107.37 (9)
C17A—N1—C2	116.81 (9)	C19—C18—C23	120.47 (12)
C17A—N1—S1	119.73 (8)	C19—C18—S1	119.61 (9)
C2—N1—S1	119.66 (8)	C23—C18—S1	119.92 (10)
C3—C2—N1	110.33 (9)	C18—C19—C20	119.50 (12)
C3—C2—H2A	109.6	C18—C19—H19	120.2
N1—C2—H2A	109.6	C20—C19—H19	120.2
C3—C2—H2B	109.6	C19—C20—C21	121.04 (12)
N1—C2—H2B	109.6	C19—C20—H20	119.5
H2A—C2—H2B	108.1	C21—C20—H20	119.5
C4—C3—O18	110.28 (10)	C20—C21—C22	118.49 (12)
C4—C3—C2	133.66 (11)	C20—C21—C24	120.77 (12)
O18—C3—C2	115.88 (10)	C22—C21—C24	120.73 (13)
C3—C4—C5	106.38 (11)	C23—C22—C21	121.19 (12)
C3—C4—H4	126.8	C23—C22—H22	119.4
C5—C4—H4	126.8	C21—C22—H22	119.4
C6—C5—C4	106.62 (11)	C22—C23—C18	119.30 (12)
C6—C5—H5	126.7	C22—C23—H23	120.3
C4—C5—H5	126.7	C18—C23—H23	120.3
C5—C6—O18	109.96 (10)	C21—C24—H24A	109.5
C5—C6—C7	133.39 (11)	C21—C24—H24B	109.5
O18—C6—C7	116.59 (10)	H24A—C24—H24B	109.5
N8—C7—C6	112.77 (10)	C21—C24—H24C	109.5
N8—C7—H7A	109.0	H24A—C24—H24C	109.5
C6—C7—H7A	109.0	H24B—C24—H24C	109.5
N8—C7—H7B	109.0	N8—C25—C26	110.98 (10)
C6—C7—H7B	109.0	N8—C25—H25A	109.4
H7A—C7—H7B	107.8	C26—C25—H25A	109.4
C25—N8—C7	109.86 (10)	N8—C25—H25B	109.4
C25—N8—C9	111.95 (9)	C26—C25—H25B	109.4
C7—N8—C9	113.15 (9)	H25A—C25—H25B	108.0
N8—C9—C10	113.10 (10)	C27—C26—C31	118.74 (12)
N8—C9—H9A	109.0	C27—C26—C25	121.66 (11)
C10—C9—H9A	109.0	C31—C26—C25	119.59 (11)
N8—C9—H9B	109.0	C26—C27—C28	120.51 (12)
C10—C9—H9B	109.0	C26—C27—H27	119.7
H9A—C9—H9B	107.8	C28—C27—H27	119.7
C11—C10—O19	109.65 (10)	C29—C28—C27	120.05 (13)
C11—C10—C9	133.17 (11)	C29—C28—H28	120.0
O19—C10—C9	117.17 (10)	C27—C28—H28	120.0
C10—C11—C12	106.75 (11)	C28—C29—C30	119.86 (13)
C10—C11—H11	126.6	C28—C29—H29	120.1
C12—C11—H11	126.6	C30—C29—H29	120.1
C13—C12—C11	106.89 (11)	C31—C30—C29	120.07 (13)
C13—C12—H12	126.6	C31—C30—H30	120.0
C11—C12—H12	126.6	C29—C30—H30	120.0
C12—C13—O19	109.34 (10)	C30—C31—C26	120.76 (13)
C12—C13—C13A	131.60 (11)	C30—C31—H31	119.6
O19—C13—C13A	119.05 (10)	C26—C31—H31	119.6
C17A—C13A—C14	117.06 (11)	C32—O3—H3	108 (4)
C17A—C13A—C13	125.36 (10)	C33—C32—O3	114.2 (4)
C14—C13A—C13	117.58 (10)	C33—C32—H32A	108.7
C15—C14—C13A	122.22 (11)	O3—C32—H32A	108.7
C15—C14—H14	118.9	C33—C32—H32B	108.7
C13A—C14—H14	118.9	O3—C32—H32B	108.7
C14—C15—C16	119.77 (11)	H32A—C32—H32B	107.6
C14—C15—H15	120.1	C32—C33—H33A	109.5
C16—C15—H15	120.1	C32—C33—H33B	109.5
C17—C16—C15	119.17 (11)	H33A—C33—H33B	109.5
C17—C16—H16	120.4	C32—C33—H33C	109.5
C15—C16—H16	120.4	H33A—C33—H33C	109.5
C16—C17—C17A	121.10 (11)	H33B—C33—H33C	109.5
C16—C17—H17	119.4		
			
O1—S1—N1—C17A	−36.74 (10)	C13—C13A—C17A—N1	−5.47 (18)
O2—S1—N1—C17A	−166.83 (9)	C2—N1—C17A—C17	68.97 (14)
C18—S1—N1—C17A	78.08 (10)	S1—N1—C17A—C17	−88.99 (12)
O1—S1—N1—C2	165.92 (8)	C2—N1—C17A—C13A	−108.37 (12)
O2—S1—N1—C2	35.83 (10)	S1—N1—C17A—C13A	93.67 (12)
C18—S1—N1—C2	−79.25 (9)	C4—C3—O18—C6	2.15 (12)
C17A—N1—C2—C3	79.15 (12)	C2—C3—O18—C6	−173.59 (9)
S1—N1—C2—C3	−122.87 (9)	C5—C6—O18—C3	−2.10 (12)
N1—C2—C3—C4	−99.88 (15)	C7—C6—O18—C3	175.43 (9)
N1—C2—C3—O18	74.60 (12)	C12—C13—O19—C10	−0.20 (13)
O18—C3—C4—C5	−1.38 (13)	C13A—C13—O19—C10	179.08 (10)
C2—C3—C4—C5	173.32 (12)	C11—C10—O19—C13	0.40 (13)
C3—C4—C5—C6	0.07 (13)	C9—C10—O19—C13	−179.30 (10)
C4—C5—C6—O18	1.27 (13)	O1—S1—C18—C19	14.28 (12)
C4—C5—C6—C7	−175.70 (12)	O2—S1—C18—C19	145.20 (10)
C5—C6—C7—N8	85.18 (16)	N1—S1—C18—C19	−100.41 (10)
O18—C6—C7—N8	−91.63 (12)	O1—S1—C18—C23	−166.03 (10)
C6—C7—N8—C25	−165.59 (10)	O2—S1—C18—C23	−35.11 (12)
C6—C7—N8—C9	68.49 (13)	N1—S1—C18—C23	79.28 (11)
C25—N8—C9—C10	131.45 (11)	C23—C18—C19—C20	−0.19 (19)
C7—N8—C9—C10	−103.75 (12)	S1—C18—C19—C20	179.50 (10)
N8—C9—C10—C11	−93.30 (16)	C18—C19—C20—C21	0.46 (19)
N8—C9—C10—O19	86.32 (13)	C19—C20—C21—C22	−0.6 (2)
O19—C10—C11—C12	−0.43 (14)	C19—C20—C21—C24	178.56 (12)
C9—C10—C11—C12	179.21 (13)	C20—C21—C22—C23	0.5 (2)
C10—C11—C12—C13	0.30 (14)	C24—C21—C22—C23	−178.67 (13)
C11—C12—C13—O19	−0.06 (14)	C21—C22—C23—C18	−0.2 (2)
C11—C12—C13—C13A	−179.21 (12)	C19—C18—C23—C22	0.1 (2)
C12—C13—C13A—C17A	−154.74 (13)	S1—C18—C23—C22	−179.60 (11)
O19—C13—C13A—C17A	26.17 (17)	C7—N8—C25—C26	68.38 (13)
C12—C13—C13A—C14	24.52 (19)	C9—N8—C25—C26	−165.03 (10)
O19—C13—C13A—C14	−154.57 (10)	N8—C25—C26—C27	−126.60 (12)
C17A—C13A—C14—C15	1.30 (17)	N8—C25—C26—C31	53.88 (16)
C13—C13A—C14—C15	−178.02 (11)	C31—C26—C27—C28	0.49 (19)
C13A—C14—C15—C16	0.15 (19)	C25—C26—C27—C28	−179.04 (12)
C14—C15—C16—C17	−0.92 (19)	C26—C27—C28—C29	−1.0 (2)
C15—C16—C17—C17A	0.22 (19)	C27—C28—C29—C30	0.8 (2)
C16—C17—C17A—C13A	1.28 (18)	C28—C29—C30—C31	−0.3 (2)
C16—C17—C17A—N1	−176.08 (11)	C29—C30—C31—C26	−0.2 (2)
C14—C13A—C17A—C17	−1.99 (17)	C27—C26—C31—C30	0.11 (19)
C13—C13A—C17A—C17	177.27 (11)	C25—C26—C31—C30	179.65 (12)
C14—C13A—C17A—N1	175.27 (10)		

### Hydrogen-bond geometry (Å, º)

*Cg*1, *Cg*3 and *Cg*5 are the centroids of the O18/C3–C6 furan, C13*A*/C14–C17/C17*A* benzene and C26–C31 phenyl rings, respectively.

**Table:**

*D*—H···*A*	*D*—H	H···*A*	*D*···*A*	*D*—H···*A*
C9—H9*A*···O1^i^	0.99	2.53	3.4057 (17)	148
C25—H25*B*···O1^i^	0.99	2.59	3.5125 (18)	155
O3—H3···O2	0.88 (7)	2.52 (6)	3.251 (3)	141 (6)
O3—H3···O18	0.88 (7)	2.47 (7)	3.097 (4)	129 (5)
C2—H2*A*···*Cg*1^ii^	0.99	2.56	3.2916 (12)	131
C16—H16···*Cg*5^iii^	0.95	2.80	3.5220 (15)	133
C20—H20···*Cg*3^iv^	0.95	2.83	3.5707 (15)	135

Symmetry codes: (i) −*x*, −*y*+1, −*z*+1; (ii) −*x*+1, −*y*, −*z*+1; (iii) *x*+1, *y*, *z*−1; (iv) −*x*+1, −*y*+1, −*z*.
